# Intestinal and Renal Adaptations to Changes of Dietary Phosphate Concentrations in Rat

**DOI:** 10.1093/function/zqad063

**Published:** 2023-11-13

**Authors:** Susana Lucea, Gema Chopo-Escuin, Natalia Guillén, Cecilia Sosa, Víctor Sorribas

**Affiliations:** Laboratory of Molecular Toxicology, Department of Biochemistry and Cell and Molecular Biology, University of Zaragoza, E50013 Zaragoza, Spain; Laboratory of Molecular Toxicology, Department of Biochemistry and Cell and Molecular Biology, University of Zaragoza, E50013 Zaragoza, Spain; Laboratory of Molecular Toxicology, Department of Biochemistry and Cell and Molecular Biology, University of Zaragoza, E50013 Zaragoza, Spain; Laboratory of Molecular Toxicology, Department of Biochemistry and Cell and Molecular Biology, University of Zaragoza, E50013 Zaragoza, Spain; Laboratory of Molecular Toxicology, Department of Biochemistry and Cell and Molecular Biology, University of Zaragoza, E50013 Zaragoza, Spain

**Keywords:** FGF23, NaPi2a, NaPi2b, Pi transport, Pi adaptation, Pi homeostasis, PTH

## Abstract

We have studied the role of the intestine, kidney, and several hormones when adapting to changes in dietary P concentration. Normal and parathyroidectomized (PTX) rats were fed pH-matched diets containing 0.1%, 0.6%, and 1.2% P concentrations. ^32^Pi uptake was determined in the jejunum and kidney cortex brush border membrane vesicles. Several hormone and ion concentrations were determined in the blood and urine of rats. Both jejunum and kidney cortex Pi transport was regulated with 5 d of chronic feeding of P diets in normal rats. Acute adaptation was determined by switching foods on day 6, which was only clearly observed in the kidney cortex of normal rats, with more statistical variability in the jejunum. However, no paradoxical increase of Pi uptake in the jejunum was reproduced after the acute switch to the 1.2% P diet. Pi uptake in the jejunum was parathyroid hormone (PTH)-independent, but in the kidney, the chronic adaptation was reduced, and no acute dietary adaptations were observed. The NaPi2a protein was more abundant in the PTX than the sham kidneys, but contrary to the modest or absent changes in Pi uptake adaptation, the transporter was similarly regulated by dietary P, as in the sham rats. PTH and fibroblast growth factor 23 (FGF23) were the only hormones regulated by all diet changes, even in fasting animals, which exhibited regulated Pi transport despite similar phosphatemia. Evidence of Pi appetite effects was also observed. In brief, our results show new characteristics of Pi adaptations, including a lack of correlation between Pi transport, NaPi2a expression, and PTH/FGF23 concentrations.

## Graphical Abstract

**Graphical Abstract:**
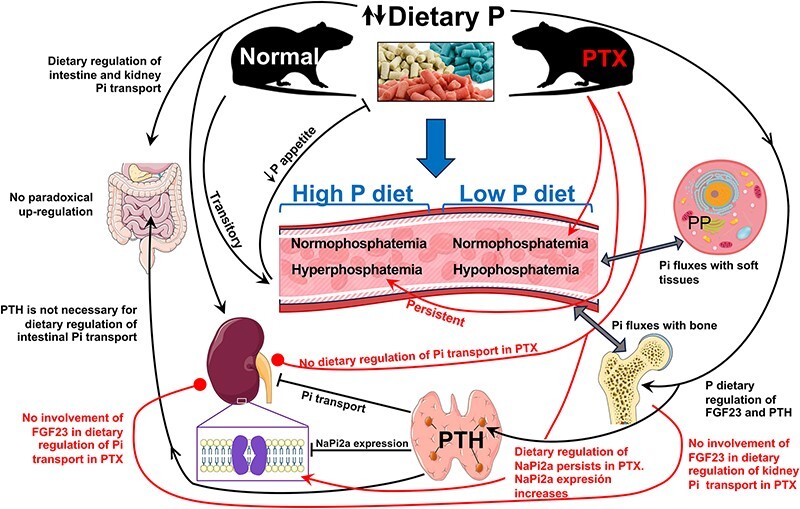

## Introduction

As an essential component of all organisms, inorganic phosphate (Pi) homeostasis is accurately maintained through the orchestrated activity of several mechanisms.^1–3^ In complex organisms, these mechanisms consist of hormonal and nonhormonal regulators that act on plasma membrane Pi transporters, which are mainly located in the proximal tubules of the kidney and in the small intestine. A modification of the abundance of the Slc34 and Slc20 family members of Pi transporters in the plasma membrane is the most relevant mechanism of adaptation to changes in dietary Pi. The main purpose of that mechanism is to keep the supply of phosphate constant.^4^, ^5^ For example, when the dietary Pi content is reduced, the increased expression of Pi transporters in the luminal membrane of the kidney proximal tubules successfully prevents the urinary loss of precious Pi. Conversely, when dietary Pi is increased, Pi transporters in the apical membrane of the proximal tubules are internalized, thereby increasing urine Pi loss and preventing the toxic outcomes of hyperphosphatemia.^6^

The many regulators of Pi homeostasis do not act independently, rather, they are interrelated and form a complex network of agents with positive and negative feedback, therefore hindering the study of Pi handling (see Table 1 in Lucea et al.^7^). The kidney is the main organ responsible for maintaining Pi homeostasis when a fast response is necessary, and the PTH and fibroblast growth factor 23 (FGF23) are the main known hormones that act acutely on the Pi reabsorption rate. However, there is strong, recent evidence of additional regulator(s) that act after an acute, high intake of Pi.^8^ In the small intestine, the paracellular route of absorption seems to be very important and proportionally related to the dietary intake of Pi,^9^ but the mechanisms of regulation, if any, are still unknown.^10^ In addition, another unclear finding is what has been observed during acute adaptation of the small intestine to a sudden increase of P intake, which was described as a paradoxical increase of Pi transport and the expression of Pi transporter NaPi2b in rat duodenum.^11^ Subsequently, in addition to the intestinal response to an acute increase of dietary P, a similar change was observed in chronic feeding when the feeding time was restricted to 4 h daily.^12^ The change in the magnitude of transport was less than in the previous work,^11^ but it affected both the duodenum and the jejunum.^12^ The abundance of NaPi2b was always higher with the 0.1% P food and was independent of the feeding time regime.^12^ The molecular mechanisms of this regulation and the intestinal responses are still unclear, but the molecular and kinetic analyses of intestinal transport, as well as the effects of pH and phosphonoformic acid, suggested the presence of new, additional Pi transport systems.

While many physiological agents are able to alter Pi homeostasis when administered to experimental animals, not all of them are presumably involved during the process of dietary adaptations because their endogenous concentrations are not modified when the dose of dietary Pi is changed.^13^ These agents include several hormones, the inositol hexakisphosphate kinases,^8^, ^14^ and the nicotinamide adenine dinucleotides NAD^+^ and NADH, which control the daily oscillations of phosphatemia.^15^ These 2 compounds have also been described as competitive inhibitors of renal and intestinal Pi transport in vitro and in vivo.^16^ However, that inhibition finally turned out to be an experimental artifact,^7^ and the role of those compounds during adaptation to dietary Pi is still unclear.

Changes in the concentrations of these physiological agents in tissues or blood can be interpreted as either a cause or a consequence of the adaptation process. In the case of PTH and FGF23, their response times are different after a high P intake^13^ and after their blood concentrations increase, but neither is fully responsible for the renal adaptation response.^8^ With respect to intestinal adaptation, the role of FGF23 as a direct modulator is unlikely, given that the expression of the co-receptor klotho is very limited and that the only significant FGF activity is that of FGF15/19.^17^, ^18^ However, the intestine contains PTH receptors that could be involved in some of the adaptation mechanisms.^19^, ^20^

In this work, we have tried to contribute to an understanding of the intestinal and renal adaptations to modifications of dietary Pi doses. We have analyzed the changes in several parameters, hormones, and nonhormone agents using several feeding strategies and fodders with feasible concentrations of Pi and using rats that were trained to eat at specific times. To better understand the role of PTH, we have also used parathyroidectomized (PTX) rats in some of the experiments, thereby confirming the predominant role of this hormone.

## Materials and Methods

### Animals and Foods

**Figure 1.**
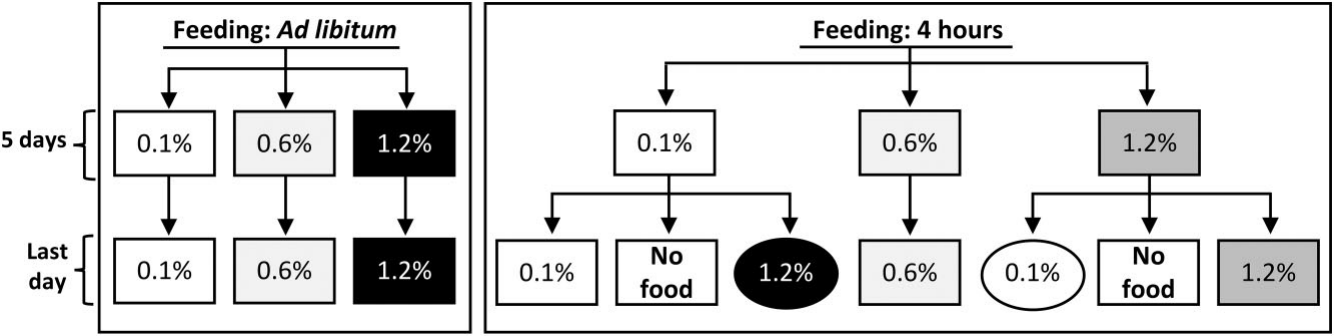
Experimental design. The outline shows the AL-fed and the 4-h restricted feeding time groups. Ad libitum feeding only includes chronic adaptations, whereas the 4-h restricted feeding regime includes the chronic and acutely adapted groups. Acute adaptation was achieved by switching the food on the last day (oval shape). Some of the animals were also euthanized on the last day before feeding to check the effect of overnight fasting. See the text for details.

For the first part of the experimentation, 2-mo-old normal male Wistar rats were obtained from Janvier Laboratories (Saint Berthevin Cedex, France). Sham-operated and PTX Wistar rats, also 2 mo old, were obtained from Envigo RMS Spain (Barcelona). All animals were cared for in accordance with European and Spanish legislation, and all procedures were approved by the Ethical Committee for Animal Experimentation of the University of Zaragoza, with authorization number PI22/21. In the first experiment, 44 nonoperated rats were used, and in the second experiment (sham versus PTX rats), 40 animals were used (see the “Statistics” section for the distribution per group). For the ad libitum (AL) groups, only 3 animals were used because this condition has been studied extensively. For the acute adaptations, 5 nonoperated rats and 4 sham and PTX rats were used in the respective experiments. To prevent the hungry bone syndrome, the PTX rats were given drinking water containing calcium gluconate (350 mg Ca^2+^/L). The feeding regime is explained in Figure 1 and in the “Results” section.

The 3 fodders used in this study were obtained from SAFE Diets (Augy, France). The concentration of P in AIN food was achieved with a combination of NaH_2_PO_4_ and K_2_HPO_4_ so that all 3 fodders had a pH of 7.2. The main components and variable differences are shown in Table 1.

**Table 1.** Major Differences in Food Composition

Components	0.1% P	0.6% P	1.2% P
AIN minerals (no KH_2_PO_4_), g/kg	35.0	35.0	35.0
K_2_HPO_4_	0	18.9	41.3
NaH_2_PO_4_	0	8.1	17.7
Sodium, %	0.29	0.29	0.44
Potassium, %	0.96	0.96	1.97
Phosphorus, %	0.10	0.62	1.23
Minerals, %	4.2	4.2	6.5
Pregelatinized cornstarch, g/kg	377.9	370.5	338.5

### Brush-Border Membrane Vesicle (BBMV) Preparation and Transport Assays

The rats were anesthetized by an intraperitoneal injection of pentobarbital. Subsequently, aortic blood samples were obtained, the jejunum and kidneys were removed, and the heart was cut for exsanguination. Membrane vesicles were prepared from kidney cortex and jejunum scrapings as described, using the classic procedure of double magnesium precipitation.^11^, ^12^, ^21^ Total ^32^P-Pi uptake was determined by rapid filtration, as described,^22^ at pH 7.4, using 0.05 m m Pi as the substrate (in the range of the *K*_m_ from the jejunum and kidney cortex BBMV) and for 10 s, which corresponds to the limit of initial velocity time. Uptake data are expressed as velocity (ie, per second).

### Biochemical Determinations

The concentrations of phosphate, calcium, and creatinine in arterial blood plasma and urine were determined using the corresponding QuantiChrom kits (DIPI-500, DICA-500, and DICT-500) from BioAssay Systems. Urine was collected for 24 h in metabolic cages, and therefore acute food switches for 4 h were not analyzed.

The plasma concentrations of several hormones were determined by ELISA using the following kits and according to the manufacturer’s protocols: rat intact PTH (Immunotopics, cat. 60-2500), FGF-23 (Kainos Laboratories, CY-4000), Rat Klotho KLOT (BlueGene, E02K0027), secreted frizzled-related protein 4 (SFRP4; Biomatik, EKU07221), matrix extracellular phosphoglycoprotein (MEPE; Cloud-Clone Corp., SEB232Ra), dopamine (Cloud-Clone Corp, CEA851Ge), rat CORT for corticosterone (Elabscience, EL-0160), rat thyroxine (T4) ELISA Kit (Cusabio, E05082r), General Calcitriol ELISA Kit (EIAab, E0467Ge), and Insulin kit (Could-Clone Corp, CEA448Ra).

Total nicotinamide adenine dinucleotide was determined with an NAD^+^/NADH Assay Kit (Abcam, ab65348) in 20 mg of kidney cortex and jejunum scrapings, following the kit’s procedure. Optical density was determined at 450 nm with a DTX 880 Multimode Detector (Beckman Coulter).

### Western Blot

Immunoblots and detection were performed using kidney cortex BBMV, as described.^7^, ^12^ NaPi2a polyclonal antibody has been characterized previously.^22^ Secondary, anti-rabbit IgG antibody (cat. 31460; Invitrogen, Carlsbad, CA, USA) was used at 1:20 000 dilution.

### Statistics

Statistical analyses of the data were performed using Prism 10 (GraphPad Software, Boston, MA, USA; www.graphpad.com) for Macintosh. In the first experiment, 44 nonoperated rats were used with the following distribution: chronically fed and AL, 3 rats per group; chronically fed for 4 h per day and euthanized before feeding time (ie, fasting), 4 rats per group; chronically fed for 4 h per day, 5 rats with 0.1% P food, 5 rats with 1.2% P food, and 3 with 0.6% P food. The 2 groups of rats acutely switched on the last day from 0.1% to 1.2% P food and from 1.2% to 0.1% P food, 5 rats per group. In the second experiment, sham- versus PTX-operated rats, 4 rats per group were used. The number of rats corresponds to the dots in the figure charts.

Data are expressed as the mean ± SEM. A normal/Gaussian distribution of data was checked for all experiments using the Shapiro-Wilk test. The significances of comparisons were determined using parametric methods, either with a *t*-test for 2 means or an analysis of variance (ANOVA) for more than 2 means. An ordinary ANOVA was used when homogeneity of variance was shown using the Brown-Forsythe and Barlett’s tests. Subsequently, difference significances of selected mean comparisons were analyzed with Fisher’s Least Significant Difference (LSD) post hoc test. When variances were not homogeneous, instead of an ordinary ANOVA, both Welch’s and Brown-Forsythe’s alternative ANOVAs were used, followed by a comparison of selected means with an unpaired *t*-test with Welch’s correction post hoc. Differences were considered significant when *P* < .05. See figure legends for details, as well as the statistical procedures used in each case.

## Results

### Experimental Design and the Consumption of Food and Phosphorus

The basic experimental design is depicted in Figure 1, similar to previous studies.^12^ Normal rats were fed for 5 d with one of the indicated fodders, containing either 0.1%, 0.6%, or 1.2% P. The foods were formulated with combinations of dihydrogen phosphate [H_2_PO_4_]^−^ and hydrogen phosphate [HPO_4_]^2−^ salts so that all 3 would have the same pH of 7.2 (see Table 1). Three groups of animals were fed AL with the corresponding food, and animals of 3 additional groups were given the same food for only 4 h per day in the morning (8:00 am to 12:00 noon). On the last day, the animals restricted to feeding for only 4 h received either the same food as the previous days, no food (ie, fasting for 20 h), or the opposite food with respect to the P concentrations, meaning that some of the 0.1% P-fed animals received the 1.2% P food, and some of the 1.2% P-fed animals received the 0.1% P food (Figure 1, oval shapes).

**Figure 2.**
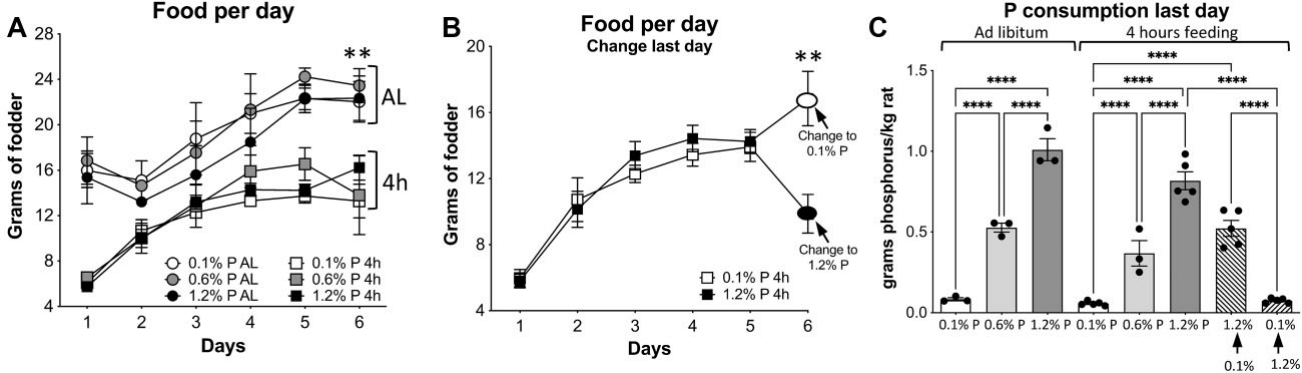
Food consumption of the experimental groups. (A) Food consumption of normal animals being fed AL versus 4-h restricted feeding. Consumption was significantly lower in the 4-h-fed animals compared to the AL-fed animals, for all 3 foods. For simplicity, statistics are only shown for the sixth day. An ordinary ANOVA (*P* < .005) was used after checking for SD homogeneity, followed by a Fisher’s LSD post hoc test for comparing the consumption of the same food with the 2 feeding patterns (AL versus 4 h). Asterisks (**) indicate ANOVA *P* < .005. Individual *P*-values for the 3 diets are as follows: 0.1% P, *P* < .005; 0.6% P, *P* < .005; and 1.2% P, *P* < .05. (B) Food consumption of 0.1% P and 1.2% P in the 4-h restricted animals, which on the last day received the opposite food in terms of P concentration, to check for acute adaptations. **P < .01 with *t*-test, 6th day. (C) Phosphorus consumption per kg of rat during the last 24 h in all rat groups. Relevant comparisons are shown, using ANOVA (*P* < .0001) and Fisher’s LSD post hoc for the indicated comparisons (see the “Materials and Methods” section): ****P < .0001.

As expected, the AL-fed animals ate more food than the 4-h restricted animals, with no significant differences between the amounts of the 3 fodders ingested within each feeding regime, namely AL and 4 h (Figure 2A). On the sixth day, however, the food switch to study the acute adaptations had a critical effect on consumption: The animals whose diet was changed from 1.2% to 0.1% P fodder ate 1.7 times more food than the animals that were switched to the 1.2% P diet after 5 d of being fed with the 0.1% P food (Figure 2B). This effect is most likely not due to an unpleasant taste of the high P diet, given that consumption had been identical for the 0.1% and 1.2% P diets during the 5 preceding days for both groups: the 4-h restricted animals and the AL-fed rats. Despite that unexpected effect, the animals that were acutely switched to the 1.2% P diet ate 0.52 g of phosphorus per kilogram of rat during the last day of the experiment (for 4 h), compared to the animals that were acutely switched to the 0.1% P diet, which consumed 0.07 g P/kg of rat (Figure 2C). Figure 2 also shows the P consumptions of the other groups and the most relevant significant comparisons.

### Effects of Adaptations on Basic Homeostatic Parameters

**Figure 3.**
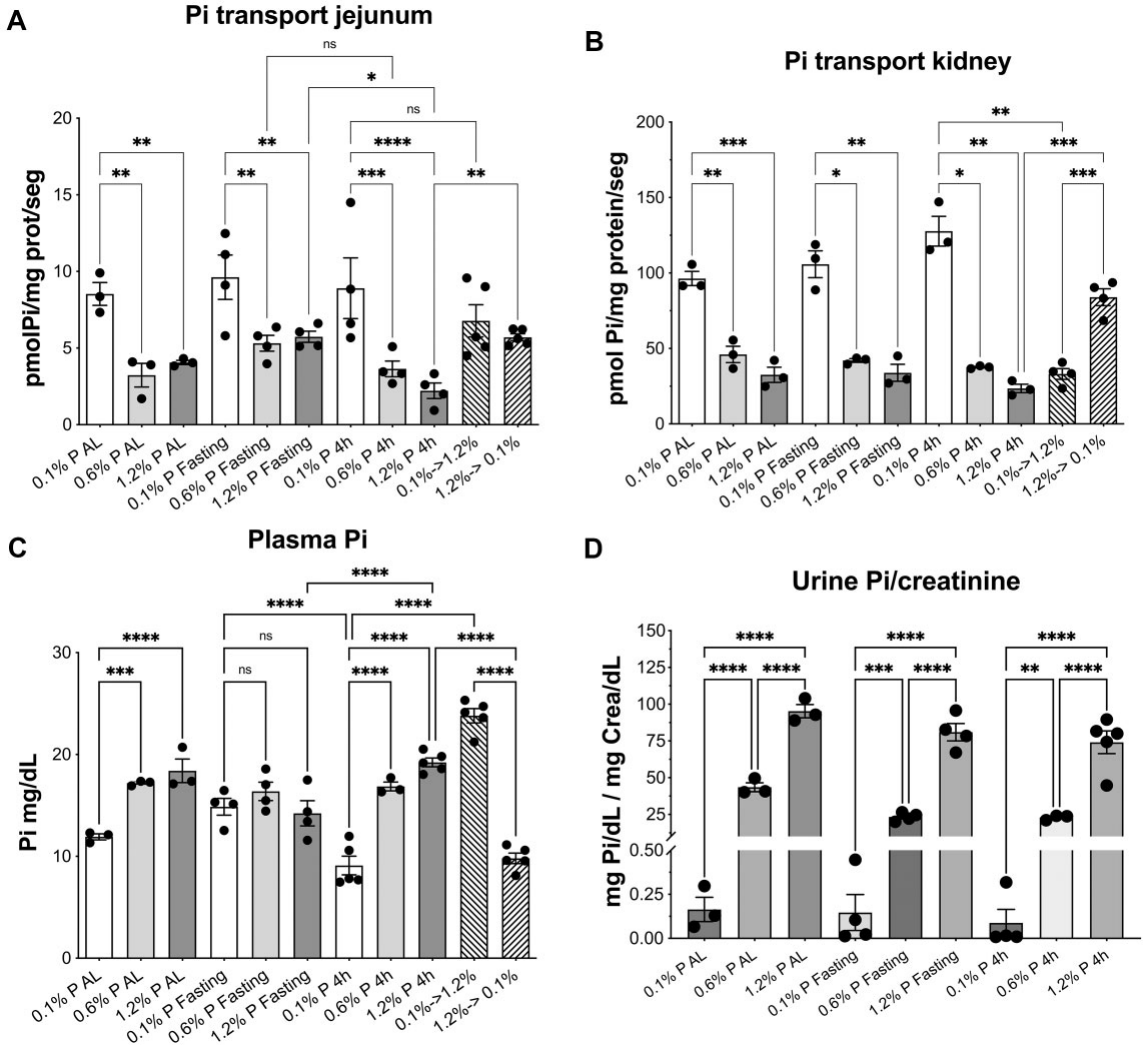
Effect of Pi diet changes on Pi transport and on blood and urine concentrations. (A) Pi transport in the jejunum BBMV from rats fed with food containing the indicated P concentration. SDs were not significantly different, and therefore an ordinary ANOVA was performed, followed by a comparison of selected means with Fisher’s LSD post hoc test (see the “Materials and Methods” section): *P < .05; **P < .01; ***P < .001; ****P < .0001. (B) Pi transport in the kidney cortex BBMV. Both Brown-Forsythe’s and Bartlett’s tests indicated significantly different SDs, and therefore an alternative ANOVA was used (Brown-Forsythe’s and Welch’s ANOVA tests, *P* < .0001), followed by a multiple comparison post hoc test with Welch’s correction (see the “Materials and Methods” section): *P < .05; **P < .01; ***P < .001. (C) Arterial blood plasma Pi concentrations in all groups of animals. Note the similar Pi concentrations in fasting animals, independently of the P diet. The ordinates measure Pi, and therefore the values are higher than just measuring phosphorus. A Brown-Forsythe’s test revealed homogeneous SDs, and therefore an ordinary ANOVA (*P* < .0001) was used, followed by a Fisher’s LSD post hoc test for multiple comparisons. ***P < .001; ****P < .0001. (D) Pi concentrations in 24-h urine (except for the 20-h fasting animals, whose urine is for 20 h) with respect to creatinine concentrations in the indicated groups. In all panels, the fasting animals refer to those euthanized on the last day just before feeding with the indicated P fodder. Statistics are as in (C), with homogeneous SDs. **P < .005.

We measured the Pi transport in the jejunum and kidney cortex BBMV, thereby confirming the described acute and chronic adaptations in the kidney to changes in dietary P concentrations (Figure 3). In the jejunum, however, previous dietary adaptation results were only partially confirmed.

As expected, the highest Pi uptake rate in the kidney was observed with the 0.1% P food, in all feeding regimes (Figure 3B). This was also the case of the jejunum during chronic adaptation with both AL and time-restricted feeding (Figure 3A), contrary to previous studies in which either an acute change to a high-Pi diet^11^, ^12^ or just feeding for 4 h per day paradoxically increased Pi transport in the duodenum and jejunum BBMV.^12^

In both the kidney and jejunum, the notable effect of the P-deprived fodder (0.1% P) on Pi transport and the nondifference between the 0.6% and 1.2% P food in both tissues could be at least partially due to the 6-fold difference in P concentration between the 0.1% P (deprived) and at 0.6% P (control) fodders, compared to the only 2-fold difference between the food at 0.6% P and 1.2% P (enriched), in addition to the fact that the P dose of the 0.6% P food already fulfills all the nutritional P needs. With respect to the acute adaptation from a low to a high concentration of P in the food and vice versa, the kidney BBMV showed the classical fast changes in Pi transport (Figure 3B). Jejunum Pi transport adaptation also occurred, and it was similar to that of the kidney but with less intensity (Figure 3A). Also, in the switch from a low to a high P fodder, Pi transport was reduced, but the effect was not enough to be significantly different from the transport in animals that were chronically fed with the 0.1% P fodder. The jejunum transport resulting from the fodder change from 1.2% to 0.1% P was, however, significantly higher than the jejunum transport in the animals that were chronically fed the 1.2% P diet.

We also analyzed the consequences of the experimental fasting of the rats used for the acute adaptations. The acute adaptation experiments required feeding the animals for only 4 h per day for several days so that, at the same starting time, they would eat the specific diet to achieve the chronic adaptation before switching the food on the last day at the same feeding time. This meant that the food was withdrawn and that the animals fasted for 20 h (from 12:00 noon to 8 am). In order to know the physiological status of the animals in these experiments just before feeding, we analyzed several parameters related to Pi homeostasis. With respect to Pi transport, panels A and B of Figure 3 show that both the jejunum and the kidney cortex BBMV from these (fasting) animals maintained the Pi transport rate adapted to the deprived, 0.1% P food, for at least 20 h, that is, the Pi transport rate was significantly higher than the rate of fasting animals that had been eating either the 0.6% or 1.2% P food. In the jejunum, however, the Pi transport rates of fasting rats eating either 0.6% or 1.2% P food were significantly higher than the corresponding rates in the animals being fed the same food for 4 h.

The purpose of adaptation to different dietary P concentrations is to keep the Pi concentration in blood plasma at a constant level through, mainly, changes in the intestinal absorption rate and through renal reabsorption/urinary excretion. To learn if that constant level was successfully achieved during adaptation, we also measured the blood plasma Pi concentration. Figure 3C shows that, as previously described by several groups, the plasma Pi concentration varied according to the P content of the food consumed in the groups of animals that were chronically fed, either AL or restricted to 4 h of feeding daily. Consequently, a normal Pi concentration was not immediately reached in the plasma after feeding, meaning that in the case of animals that were fed for 4 h only. For example, the animals that were fed the 0.1% P food showed a lower plasma P concentration compared to the animals that were fed the 1.2% P food. Rats that are fed AL mainly eat during the night, meaning that there is a longer time span until blood sampling and less pronounced differences in plasma Pi concentration. In fact, after 20 h of fasting, that is, when the time span from eating to blood collection is maximal, differences in the plasma Pi concentrations among the 3 groups were no longer observed, even when the Pi transport rates maintained the adaptations to the low and high Pi concentrations in the food, as shown in Figure 3A and B. Notably, while all 3 plasma Pi concentrations are similar in fasting animals, when they are fed again with the corresponding foods for 4 h, 3 different responses are observed in the plasma Pi (Figure 3C, which compares fasting and being fed for 4 h): The plasma Pi concentration in rats fed with the 1.2% P diet increases significantly, as expected, while the plasma Pi concentrations remain very similar in the rats fed with the 0.6% P diet, and curiously, a significantly lower plasma Pi concentration is observed in the rats fed with the 0.1% P diet.

The plasma creatinine concentrations were similar in all groups, therefore discarding the possibility that the changes in the renal filtration rates could affect plasma Pi levels. Consequently, the renal excretion of Pi was also determined for 24 h (Figure 3D). Unlike in blood plasma, the concentration of P in urine at 24 h varied dramatically according to the exposure to dietary P. Notably, despite a similar transport rate in the renal BBMV of the rats that were fed 0.6% and 1.2% P, the urine of the animals that were fed 1.2% P contained double the concentration of Pi than the urine of the animals that were fed with 0.6% P. Therefore, unlike what happens to the renal Pi transport rate, the concentration of Pi in urine correlated perfectly with the amount of P eaten by the animals.

### Changes in the Concentration of Pi Homeostasis Regulators

**Figure 4.**
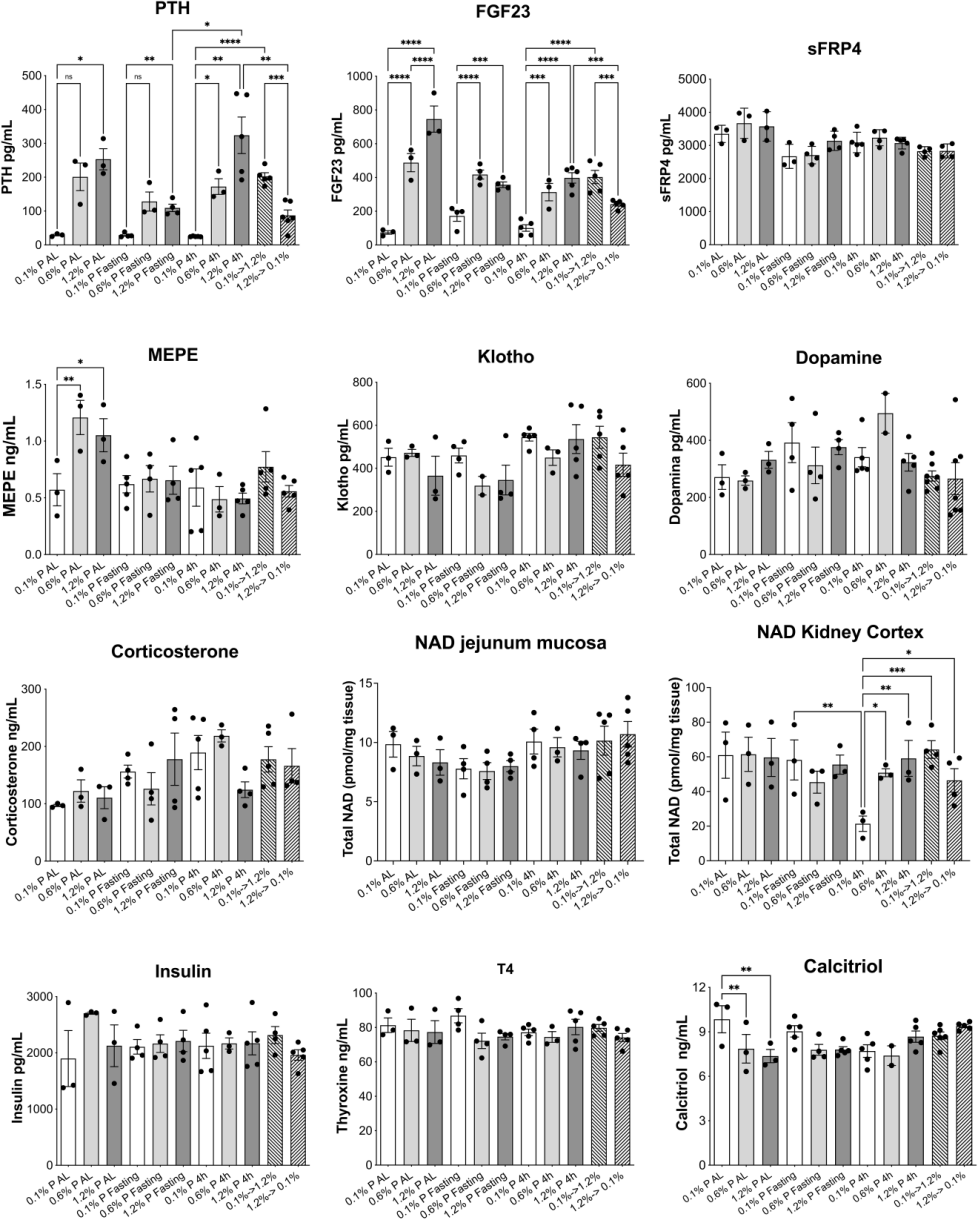
Effect of dietary Pi concentration changes on the levels of regulators of Pi homeostasis. Plasma FGF23 and PTH were the most affected hormones, with significant responses to the different diets in most of the groups, but only relevant statistical comparisons are shown. MEPE was also increased in the chronic AL-fed rats only eating 0.6% and 1.2% P diets, compared to 0.1 P food. Significant changes are shown in total NAD^+^/NADH in the kidney cortex of 4-h fed rats and in plasma calcitriol of the AL, Pi-deprived rats. In PTH, significances of differences were determined with alternative ANOVAs (*P* < .0001) because SDs were not homogeneous. The multiple comparison was done with unpaired *t*-test and Welch’s correction: *P < .05; **P < .01; ***P < .0005; ****P < .0001. Ordinary ANOVAs were performed for all other regulators, which showed homogenous SDs. Other than FGF23 (*P *< .0001), some significances were only observed for MEPE (*P* < .01), total NAD in the kidney cortex (*P* < .05), and calcitriol (*P* < .005). A Fisher’s LSD post hoc was used for selected comparisons: *P < .05; **P < .01; ***P < .001; ****P < .0001.

We also analyzed the likely changes in the concentrations of hormones and other agents known to control or modify Pi homeostasis in response to different exposures to dietary Pi (Figure 4). The hormones most affected by the dietary Pi changes were PTH and FGF23, which responded similarly to the experimental maneuvers, as expected. Their plasma concentrations increased with the dietary Pi concentration, even switching from the 0.6% P to the 1.2% P diets. However, in this case, the differences were not all significantly different, with the exception of PTH in the 4-h restricted animals. In Pi-deprived animals (0.1% P diet), the concentrations of PTH and FGF23 were extremely low, in either the AL or the 4-h feeding regime.

The rats that were subject to the acute change of food from 0.1% to 1.2% P showed a marked increase in both PTH and FGF23 compared to rats given the 0.1% P diet for 4 h per day. In rats whose food was switched from 1.2% to 0.1% P, the plasma concentrations of PTH and FGF23 were lower than those chronically fed with the 1.2% P food but were still higher than those chronically fed with P-deprived food (0.1% P), most likely as a consequence of the half-life of the hormones. Notably, despite this moderate decrease of PTH and FGF23 concentrations in the acute change to 0.1% P food, both the jejunum and renal transports of Pi had already increased compared to the chronic 1.2% P diet (Figure 3A and B).

Also very notably, in the animals that were subject to fasting for 20 h, the differences in the plasma concentrations of PTH and FGF23 among the 3 dietary groups remained. PTH concentrations in the fasting animals adapted to the 0.6% and 1.2% P diets were similar but reduced, especially in the case of 1.2% P diets, compared to the corresponding animals being fed for 4 h (Figure 4). Despite the reduction, the PTH levels were still higher than in the animals that were chronically adapted to the 0.1% P food in any condition, while similar plasma P concentrations remained for all 3 diets in the fasting animals (Figure 3C). The data dispersion of the PTH values in the 0.6% P diet of the fasting and AL-fed animals prevented a significance of the difference with 0.1% P diet, despite the higher means.

With respect to FGF23, the values in the fasting animals remained similar to the corresponding FGF23 concentrations in the nonfasting animals fed for 4 h (Figure 4). Maximal levels were obtained in the AL-fed rats with eating the 0.6% and 1.2% P foods.

The abundance of MEPE only increased significantly with the high Pi diets in the animals that were fed AL. No changes in other phosphaturic hormones were observed, including SFRP4, klotho, dopamine, and corticosterone concentrations. We also quantified NAD^+^/NADH in the jejunum mucosa and kidney cortex of all groups of animals, with no significant changes in the jejunum. In the kidney cortex, however, a low concentration was found in the animals that were fed for 4 h with the 0.1% P food, and the tissue concentration recovered in the animals that were subjected to the acute change to the 1.2% P food.

Regarding hormones that retain Pi in the body and reduce urinary loss, no changes were observed in insulin or thyroid hormone blood plasma concentrations, and only calcitriol was significantly increased by 25% in the animals that were fed AL with the P-deprived food.

Given that the nicotinamide nucleotides did not change in the jejunum tissue and that FGF23 seemed to act only in the intestine through changes in circulating vitamin D (see the “Introduction” section), we subsequently focused on the roles of PTH using PTX rats.

### Food and Phosphorus Consumption in PTX Rats

**Figure 5.**
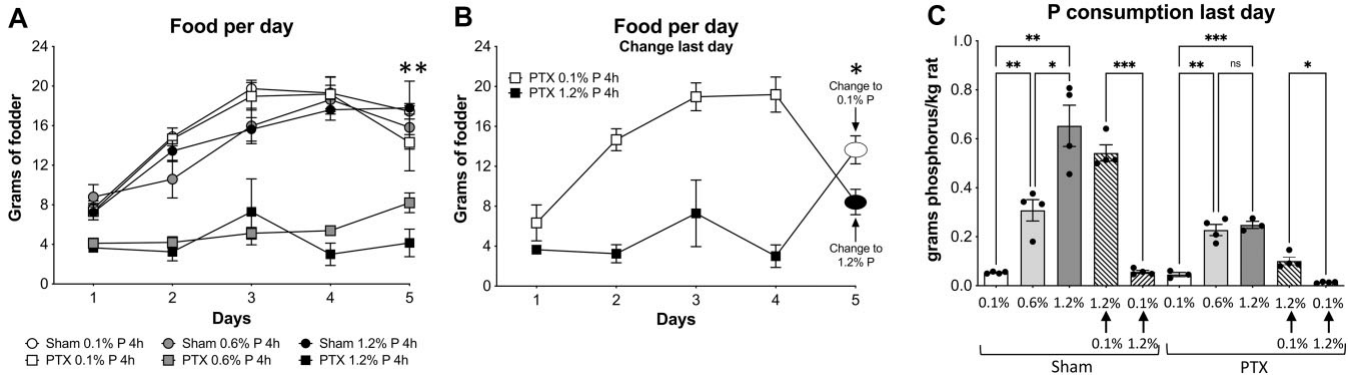
Food consumption and P exposure in sham versus PTX rats fed for 4 h per day. (A) Food consumption of sham versus PTX animals with the 3 P concentrations. PTX rats eating the 0.1% P food ate a similar amount as the sham-operated animals. An ordinary ANOVA was used for comparisons on the 6th day (**P < .005), and Fisher’s LSD post hoc test revealed that PTX rats ate less 0.6% P (*P* < .05) and 1.2% P foods (*P* < .0005) than the corresponding sham rats. (B) Food consumption of the 4-h restricted PTX animals, which on the last day received the opposite food in terms of P concentration. *P = .033 with a *t*-test for the last day comparison. (C) Phosphorus consumption per kg of rat in all groups during the last 24 h. The most relevant comparisons are shown, using an alternative ANOVA (*P* < .0001; SDs significantly different from Brown-Forsythe’s test) and the post hoc *t*-test with Welch’s correction for the indicated comparisons: *P < .05; **P < .01; ***P < .001.

In order to reduce the number of animals, no AL-fed groups were used with PTX animals. PTX rats that were fed with the 0.6% and 1.2% P foods ate a low and constant quantity of grams of fodder per day, that is, the amount of food consumed did not increase significantly during the 5 d of experimental feeding (Figure 5A).

This was not the case of the sham animals that were fed at any concentration of P or of the PTX rats eating the 0.1% P food: The food consumption of this P-restricted group was similar to that of the sham-operated rats, including the fact that food consumption increased during the first 3 d, as observed in the sham animals. As in the case of the nonoperated rats (Figure 2A), the sham-operated rats consumed similar amounts of food, regardless of the P content (Figure 5A). During the acute adaptations, however, fodder consumption by the PTX rats that had been fed with 1.2% P fodder increased on the last day when they were fed with 0.1% P (Figure 5B). Conversely, in the PTX rats chronically fed with 0.1% P fodder, the consumption dropped significantly when they received the 1.2% P food, similarly to the nonoperated rats (Figure 5A versus Figure 2A). The consumption of phosphorus on the last day by all groups of sham and PTX rats is shown in Figure 5C, and it is related (but not proportional) to the concentration of P in the food, even though the PTX rats on the 0.6% and 1.2% P diets ate less fodder.

### Pi Handling in PTX Rats

**Figure 6.**
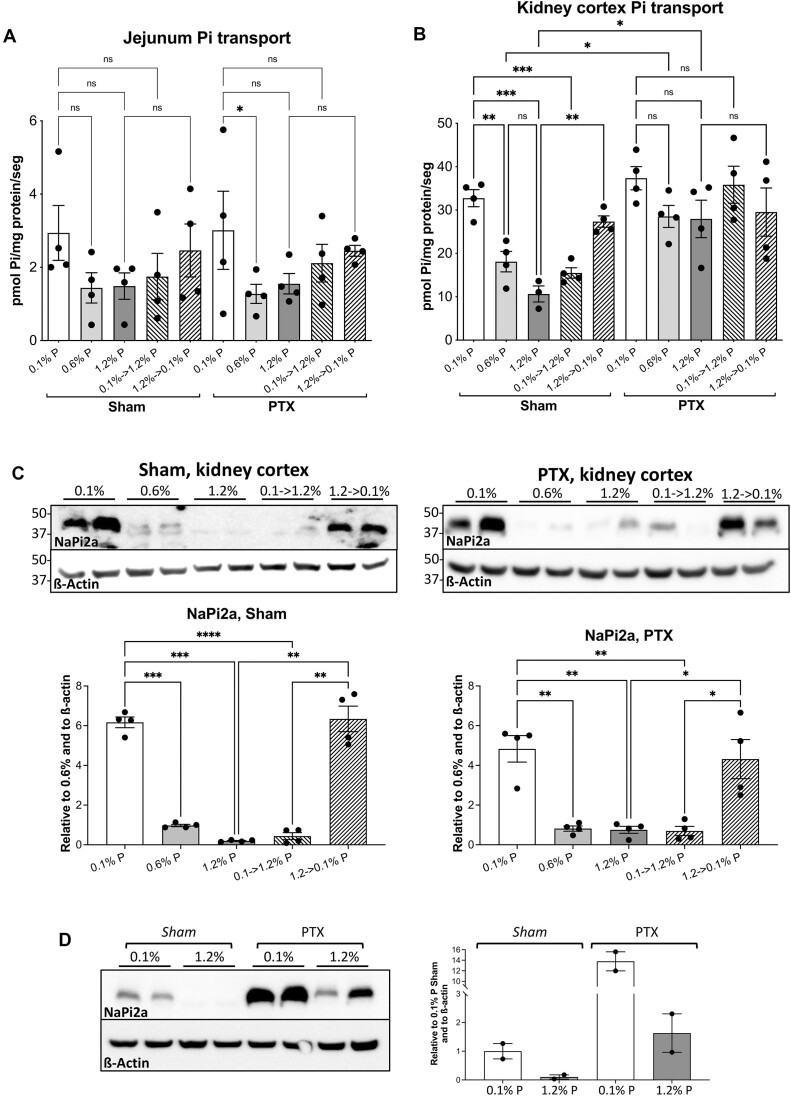
Effect of dietary phosphate and feeding regime on Pi handling in the jejunum and kidney cortex BBMV of sham versus PTX rats. (A) Effect on Pi transport in the jejunum BBMV. No paradoxical adaptations are observed, and the PTH effect is absent. An ordinary ANOVA was used, followed by a comparison of selected means with Fisher’s LSD post hoc test (*P < .05). (B) Pi transport in the kidney cortex BBMV. In contrast to sham rats, not only were no acute adaptations in PTX animals observed, but the differences in chronic adaptations were very small due to the high transport rate in BBMV from animals eating the 0.6% P and 1.2% P foods, in comparison with the sham animals. An alternative ANOVA was performed (*P* < .001) using Brown-Forsythe’s and Barlett’s tests, with selected comparisons using unpaired *t*-tests with Welch’s correction (*P < .05; **P < .005; ***P < .001). (C) Representative immunoblots of the NaPi2a transporter using aliquots of the same BBMV preparations from sham and PTX rat kidney cortex used in (B). The quantifications of band densities are shown below the immunoblots, of 2 different western blots. Density values were normalized per ß-actin and are shown relative to the 0.6% P diet. The statistical analysis was done as in (A) and (B), with *P < .05; **P < .01; ***P < .0005; and ****P < .0001. (D) Comparison of NaPi2a abundance between sham and PTX rat kidney cortex BBMV. No statistical comparison is shown because only 2 bands are used per condition. The signals of NaPi2a in the 0.1% P samples from sham rats were 10 times denser than the bands from sham rats being fed 1.2% P food. In PTX rats, there was an 8.4-fold difference in NaPi2a abundance between the 0.1% and 1.2% P foods. When comparing sham and PTX rats, the samples from PTX rats being fed the 0.1% P food were 13.8 times denser than those of sham rats, and the expression in PTX rats eating the 1.2% P food was 16 times higher than the BBMV from sham rats eating the same diet.

Pi transport was determined in BBMV from the sham and PTX rats. Using jejunum BBMV, Pi transport behaved very similarly in the sham and PTX rats, including the presence of chronic and acute adaptations to a low or high diet (Figure 6A), as observed in Figure 3A. In this second experiment, however, the differences were only significant in the case of PTX rats adapted chronically to a P-deprived diet (0.1% P).

In contrast to the jejunum, Pi uptake in the kidney cortex BBMV revealed clear differences between the sham and PTX rats (Figure 6B). The sham animals behaved as the nonoperated animals for both chronic and acute adaptations to dietary P concentration changes (see Figure 3B, 4-h feeding time restriction). The PTX rats, however, failed to adapt acutely to the concentration of P in their diet. In chronically adapted PTX rats, small but significant differences were observed between the animals eating the 0.1% P diet compared to either the 0.6% or the 1.2% P diets. The reason for this minimal difference was the high transport rate in BBMV in the PTX rats that were chronically eating the 0.6% and 1.2% P diets compared to transport in the sham rats, while transport in the case of the animals eating the 0.1% P diet was similar in both the sham and PTX animals. Therefore, PTH was completely necessary not only for acute adaptations to the high and low P diets but also for keeping a low Pi transport rate in the kidneys of animals that were chronically fed the 0.6% or 1.2% P foods.

The strikingly imperfect regulation of Pi transport in the kidney of PTX rats led us to analyze the expression of the main Pi transporter, NaPi2a (Figure 6C). In the sham animals, we found the well-known and highly correlated changes between Pi transport and NaPi2a protein expression. Unexpectedly, in the case of the PTX animals, the changes in NaPi2a expression were very similar to those in the sham rats, meaning that NaPi2a protein abundance varied inversely to the concentration of Pi in the diet. Therefore, NaPi2a expression in the PTX rats was not correlated with the expressed Pi transport shown in Figure 6B. When the expression of NaPi2a was compared between sham and PTX rats in the same blot (Figure 6D), there was once again evidence of the absence of correlation to Pi transport: The expression of NaPi2a was 13.8 times higher in PTX rats eating the 0.1% P food than in the sham rats eating the same fodder, whereas Pi transport was similar. Also, NaPi2a protein was 16 times more abundant in the kidney BBMV of PTX rats that ate the 1.2% P food, in comparison with the sham rats eating the same 1.2% P diet, while Pi transport was only 2 times higher in PTX rats than in sham rats, approximately.

### Blood Plasma and Urine Concentrations of Pi in PTX Rats

**Figure 7.**
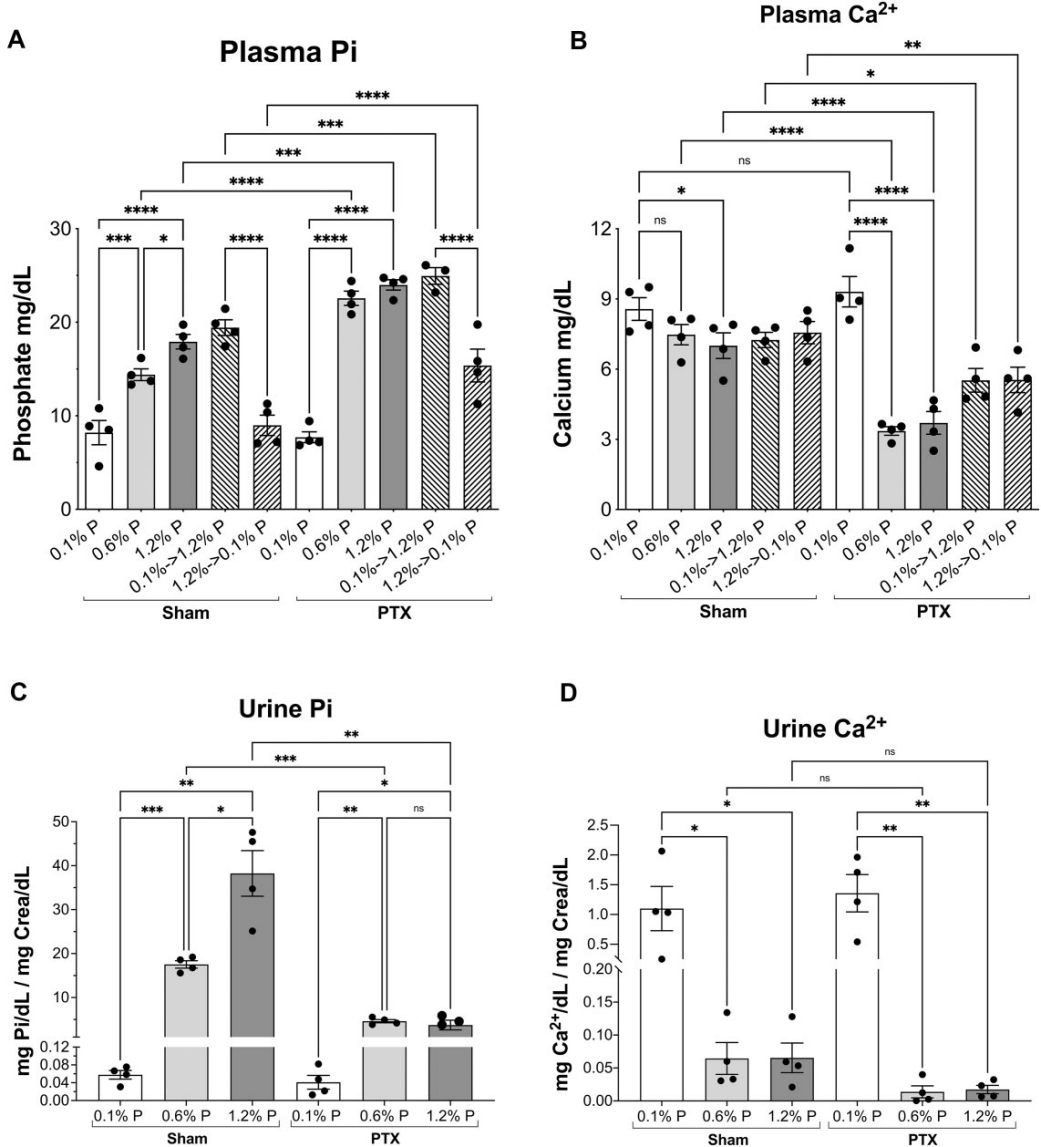
Effect of dietary P content on blood plasma and urine Pi and Ca^2+^ concentrations in PTX rats. (A) Effect on plasma Pi concentrations in sham and PTX rats. (B) Effect of dietary P content on plasma Ca^2+^ concentrations. In (A) and (B), comparisons are made using an ordinary ANOVA (*P* < .0001) since Brown-Forsythe’s test showed variance homogeneity. Multiple comparisons of selected means (all of them are shown) were done with uncorrected Fisher’s LSD test: *P < .05; **P < .01; ***P < .001; ****P < .0001. (C) Effect of dietary P dose on 24-h urinary Pi excretions in sham and PTX rats. (D) Effect on 24-h urinary excretion of Ca^2+^. In (C) and (D), no acute adaptation groups are shown because the data correspond to the 24-h collection of urine in metabolic cages. Significances of comparisons in (C) and (D) were analyzed with an alternative ANOVA for urine Pi and calcium, and an unpaired *t*-test with Welch’s correction was used for the comparisons of selected means: **P < .01; ***P < .0005; ****P < .0001.

Regarding the plasma Pi concentration (Figure 7A), there were no relevant differences between sham-operated and nonoperated rats (Figure 3C). In the PTX rats, the plasma Pi concentrations increased in all groups compared to the corresponding sham rats, with the exception of the animals that were chronically fed the 0.1% P fodder. For example, the plasma Pi concentration of the PTX rats eating the 0.6% P diet was 1.6 times the plasma Pi concentration of the sham rats eating the same food. This increase was also observed in the PTX rats chronically eating the 1.2% P food and during the 2 acute adaptations (ie, from 0.1% to 1.2% and from 1.2% to the low, 0.1% P diets).

We also determined the concentration of ionized calcium in the plasma of the sham and PTX animals (Figure 7B). The sham rats had the same Ca^2+^ concentration in plasma, regardless of the P content of the food they had eaten and regardless of the acute switches in foods. In the case of the PTX rats, however, and despite the calcium added to the drinking water, only the rats eating the low, 0.1% P food had a similar calcium concentration as the corresponding sham-rat group. Yet in the rats eating the 0.6% P and the 1.2% P foods, the plasma calcium concentrations in the PTX animals dropped to half the concentrations in the sham rats. This drop was also observed in the rats that were subjected to the acute adaptations (from high to low P concentrations in the diet, or vice versa), but the reduction was only 25%, approximately.

The 24-h urinary excretion of Pi and calcium was also determined in the sham and PTX rats (Figure 7C). As expected, the excretion of Pi was very high in the animals eating the 0.6% P diet, and with the 1.2% P food the excretion was double in the sham rats. In the PTX rats eating the 0.6% P food, the urinary excretion of Pi was 3.8 times less than that of the sham rats. The Pi excretion was similar in the PTX rats fed with either the 0.6% P or the 1.2% P diet, showing a 10-fold reduction in the very high P diet (1.2% P) and the absence of PTH compared to the sham rats. Calcium excretion was high and similar in both the sham and the PTX rats eating the deficient P diet (0.1%), and it was very low but similar in animals eating the 0.6% and 1.2% P foods. With these 2 diets, however, the PTX rats excreted 4-5 times less calcium than the sham rats, but the differences were not significant.

### Homeostatic Regulators of Pi in PTX Rats

**Figure 8.**
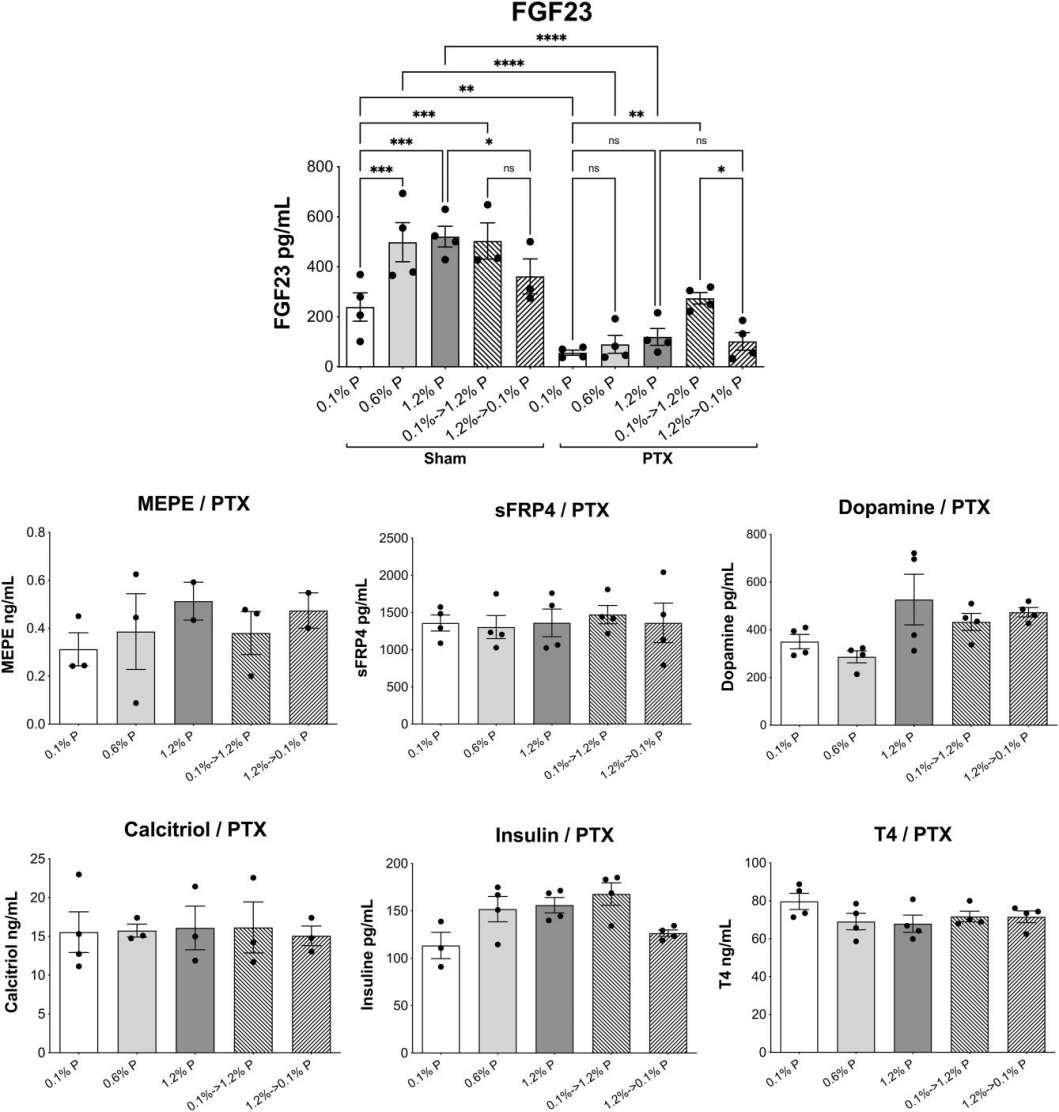
Effect of dietary P content on phosphaturic and Pi-conserving hormones, in PTX rats. Sham rats were only analyzed for FGF23. PTH hormone was not detected in PTX rats. Major changes in blood plasma were mainly observed for FGF23. The statistical analysis was performed with an ordinary ANOVA for FGF23 (*P* < .0001), MEPE, sFRP4, calcitriol, insulin, and T4. Only for FGF23, the multiple comparisons were performed with uncorrected Fisher’s LSD test because it was the only hormone significantly modified. In the case of dopamine, an alternative ANOVA also did not find significant differences. *P < .05; **P < .01; ***P < .001; ****P < .0001.

The levels of several hormones were also analyzed in the blood plasma of the PTX rats after being fed with the different foods. PTH was not detected in these animals, thereby confirming the complete PTX condition. MEPE, sFRP4, dopamine, calcitriol, insulin, and thyroid hormone were analyzed in the PTX rats, and there were no significant changes in blood plasma concentrations at any of the Pi concentrations or in any of the feeding regimes, therefore behaving as the nonoperated rats (Figure 8). Changes in the plasma FGF23 concentration were compared between the sham and PTX rats. The sham-operated rats showed FGF23 plasma levels and changes that were similar to the nonoperated rats (Figure 4). In sham rats, the difference between both acute adaptations did not reach significance, as it did in the nonoperated rats. However, in both nonoperated and sham rats, every acute adaptation was significantly different in comparison with the original state: The FGF23 concentration after the acute switch from 0.1% to 1.2% P was significantly higher than during the chronic 0.1% P situation; and with the acute change from 1.2% to 0.1% P, the FGF23 plasma concentration was significantly lower than in the chronic 1.2% P situation. In the PTX rats, the FGF23 levels were lower than in the sham rats, even in the animals that were chronically fed the 1.2% P fodder, with 4.3 times less FGF23 in their blood plasma than the sham rats. In fact, we did not observe significant differences among the PTX rats that were chronically eating the 0.1%, 0.6%, or 1.2% P diets (Figure 8). In the case of acute adaptation to a high-Pi diet (1.2% P), the blood plasma FGF23 concentration increased significantly compared to the starting, chronic 0.1% P condition. The FGF23 level after the acute change to the P-deprived diet was, however, not different than in the initial, chronic 1.2% P diet condition, but it was significantly different from the opposite acute adaptation.

## Discussion

In this work, we have analyzed the homeostatic adaptation of rats to different concentrations of phosphorus in their diets, either at constant (chronic) feeding with the same diet (with or without restriction of feeding time) or after changing the diet to another fodder with a very different concentration of phosphorus on the last day of the experimental feeding. Such adaptation phenomena seek not only to guarantee a sufficient supply of Pi but also to prevent dangerous increases of Pi in the blood plasma, which is especially relevant if such an increase were long-lasting, such as when urinary excretion is compromised. The ongoing publications of several groups show that these adaptions are very complex physiological processes that are consistently initiated through the involvement of phosphate sensors. This work was initially designed to study the paradoxical acute adaptations of the intestine to different concentrations of Pi in the diet,^11^, ^12^ but the unexpected findings led us to adjust the course of the experimentation. Below, we discuss the most relevant new knowledge from our work, according to the topic.

First, regarding adaptations to dietary Pi concentrations, we have confirmed and expanded upon several of the well-known changes that occur during adaptation to a low or a high Pi concentration in food. These changes include transport rates, urinary excretion, and the plasma concentrations of Pi, as well as the plasma levels of PTH, FGF23, and other hormones. In this work, we have focused on the transport of Pi in the kidney and the small intestine while setting aside the important paracellular route of intestinal absorption.^1^, ^23^ Nevertheless, it is important to keep in mind that, even if the paracellular route seems to be nonregulated,^10^ a percentage of each increase in plasma Pi concentration as a consequence of a high P diet is, most likely, due to paracellular absorption.^24^ As noted in the “Introduction” section, all known mechanisms for the control of Pi homeostasis act on Pi transporters, that is, on Pi transport. The absence of a known regulatory mechanism of paracellular intestinal absorption of Pi can be explained because this route is passive and nontransporter mediated. It is therefore missing the affinity (*K*_m_) and saturation (*V*_max_) kinetic characteristics,^25^ which are the traditional targets for homeostatic Pi regulators, mainly *V*_max_ or, more specifically, the number of active transporters (aside from the transport rate).

In the case of Pi deprivation (ie, 0.1% P in fodder), the adaptation response consists in a Pi transport rate increase in the intestine and the kidney to maximize the intestinal absorption and renal reabsorption of Pi (Figure 3A and B). Also, the plasma levels of the 2 main phosphaturic hormones, FGF23 and PTH, decrease to a minimum in either chronic Pi consumption or after 4 h of acute Pi deprivation (Figure 4). In mice, the reduction in the plasma concentration of FGF23 in response to a low-Pi diet is slower, given that it has not been described even after 8 h.^26^ Renal adaptation to a low-Pi diet in mice also requires a minimal dose of calcium,^27^ an effect that could be related to PTH because, in the absence of calcium, the plasma PTH concentration increased. In the PTX rats, the plasma FGF23 concentration also increases after the acute switch from a chronic 0.1% P diet to a 1.2% P diet (Figure 8), but Pi transport is not significantly altered either in the jejunum or in the kidney (Figure 6A). During chronic feeding with any of the 3 foods, however, the concentration of FGF23 in the PTX rats is not modified. This finding shows not only that FGF23 does not compensate for the absence of PTH but that the concentration of FGF23 in plasma is even significantly lower in PTX rats than in sham rats, when the Pi plasma concentration is, however, higher (Figure 7A). This strongly coincides with previous studies showing that FGF23 depends on the presence of PTH,^28^ and it confirms the importance of PTH in Pi homeostasis control.^13^

In the case of high Pi intake, the urine excretion of Pi also increases due to the reduced renal Pi transport (ie, reabsorption, Figure 3B), but this is not sufficient to prevent a temporary increase in the plasma Pi level. For example, plasma Pi doubles in animals chronically eating the 1.2% P diet for 4 h compared to the 0.1% P diet, as well as during the acute change from 0.1% to 1.2% P (Figure 3C). After 20 h of fasting, however, the plasma Pi concentrations are similar among the 3 diets (fasting group, Figure 3C). Nevertheless, the concentrations of PTH and FGF23 are still higher in these fasting animals fed with 0.6% or 1.2% P food compared to the 0.1% P diet, as shown in Figure 4 (in the case of PTH, the difference with respect to 0.6% P did not reach significance because of the dispersion of these specific data), and similarly, Pi transport in the jejunum and the kidney cortex remained increased in the fasting animals that had been fed with the 0.1% P food, despite normophosphatemia (Figure 3A-C). This means that neither the Pi transport rate nor the concentrations of phosphaturic hormones depend directly on the plasma Pi concentration, in agreement with a previous mathematical model of renal Pi adaptation based on a 100% (2x) change of Pi plasma concentration and transport rates during acute adaptation.^29^ Similarly, the changes in plasma concentration of Pi (2x) compared to the changes in the digestive lumen (6x-12x of the 0.6%-1.2% P foods compared to the 0.1% P food) also agree with the view that it is not the plasma concentration of Pi that signals to the kidney that it must adapt to dietary Pi changes, rather it is the Pi concentration in the intestine, according to the indicated model^5^ and a previous work evidencing a similar signaling axis.^30^

The normophosphatemia of fasting animals subjected to chronic feeding for 4 h per day is lost when these animals are fed again for 4 h with either the 1.2% or the 0.1% P food (Figure 3C): The plasma Pi concentration is temporarily increased with the 1.2% P diet, it remains unaltered if the 0.6% P food is given, or it once again decreases with the deprived 0.1% P food. In the case of the 1.2% P and 0.6% P diets, excess Pi is excreted in the urine due to the sustained, decreased Pi transport in the kidney (reabsorption; Figure 3B), with the help of the reduced intestinal Pi transport (Figure 3A) and the high levels of the phosphaturic hormones PTH and FGF23 (Figure 4). The hypophosphatemia observed in the animals after eating the 0.1% P food for 4 h, having been normophosphatemic before being fed, cannot be caused by the urinary loss of precious Pi because Pi transport remains upregulated in the kidney because the loss of urine Pi is minimal and because phosphaturic hormones continue to be deeply downregulated. Consequently, it seems that Pi quickly redistributes to soft tissues, most likely as intracellular polyphosphate stores.^31^ Such an influx has been related to hyperinsulinemia after feeding in human patients,^32^ but it is not apparent in our animals (Figure 4). Clearly, this major Pi flux unrelated to intestinal absorption or the renal handling of Pi homeostasis deserves further research.

In the case of animals with free access to food (no feeding time restriction), the plasma Pi concentration of the rats eating the 0.6% and 1.2% P food was only 1.4 times higher than that of the rats eating the 0.1% P food, most likely because both ingestion and renal excretion extended over a longer period of time (and mainly overnight).

Acute feeding with the 1.2% P food also increased the plasma PTH and FGF23 concentrations within 4 h (Figure 4). This FGF23 increase was not observed in a previous work when Pi was administered by intravenous infusion or oral gavage,^13^ but it did increase in a very elegant study that also used PTH-deficient mice with inhibited FGF23 signaling.^8^ In that study, the authors concluded that signals other than PTH and FGF23 were contributing to renal adaption to dietary Pi because the Pi transporters were still regulated by the dietary Pi content. While we have observed the same unexpected finding using the PTX rats, including the downregulation of the NaPi2a protein,^8^ we have observed an even more surprising finding, which is the fact that, despite such NaPi2a regulation by the dietary Pi content, Pi transport in the kidney cortex BBMV is not acutely regulated in the PTX rats and is only minimally regulated in chronic feeding with the same diets (Figure 6B). Furthermore, while the content of NaPi2a does increase in the PTX animals compared to the sham rats (Figure 6D), renal Pi transport does not increase (Figure 6B). These findings consequently suggest that, in the kidney cortex of animals without PTH, Pi transport does not depend on the simple relative abundance of the main Pi transporter, NaPi2a. All the other analyzed hormones were not modified, or the changes were minimal and nonrelevant for the purpose of the study (Figures 4 and 8). Therefore, further research is necessary to understand these findings: The noncorrelation between Pi transport and NaPi2a expression could be due to the accumulation of Pi transporters in nonfunctional domains in the absence of PTH, therefore impeding internalization; it could be due to the interaction with proteins containing PDZ domains that might alter the normal regulation of the transporter or internalization in the absence of PTH;^33^ it could be due to the role of membrane fluidity,^34^ etc. The findings presented here also open up new possibilities to understanding the effects of PTH in the renal handling of Pi homeostasis.

Second, our results regarding intestinal Pi transport that show no paradoxical regulation also merit some discussion. In 2009, it was reported that, in rats that were acutely switched from a low- to a high-Pi diet and that were also subject to similar overnight fasting followed by feeding for 4 h in the morning, there was an increase in both sodium-dependent Pi transport and the expression of NaPi2b protein abundance in the duodenum, therefore causing an increase in serum Pi.^11^ Later, our group extended this observation to the jejunum, observing that Pi uptake depended on the feeding regime^12^: When feeding chronic and AL, uptake was higher in the Pi-deprived rats (0.1% P), whereas if feeding was chronic but for only 4 h in the morning, higher uptake was observed in 1.2% P-fed rats. Consequently, an acute switch from low to high Pi in the diet, which only requires 4 h of daily feeding, also increased the Pi uptake in the duodenum and jejunum BBMV. Nevertheless, during either chronic or acute adaptations to a low-Pi diet, and independently of feeding regime, the NaPi2b protein abundance was always higher in the 0.1% P-fed rats. In this work, we tried to clarify these paradoxical divergences depending on the feeding regime, as well as clarify the direct or indirect role of PTH in these adaptations. Surprisingly, in this work and as Figures 3A and 6A summarize, the behavior in the intestine and the kidney was similar either for chronic adaptations AL or for the 4-h feeding restriction, and the animals fed with the 0.6% or 1.2% P diets always exhibited a lower Pi uptake in the jejunum BBMV (and the kidney cortex) than the animals fed with the 0.1% P food. The same behavior was observed in the PTX rats (Figure 6A). Moreover, the acute adaptation from a 0.1% to a 1.2% P diet also caused an incomplete reduction of Pi uptake. To our knowledge, the only modification in this work with respect to previous studies has been the composition of the food. In former works, the concentration of Pi in the food had been adjusted using different amounts of the acid, dihydrogen phosphate (monosodium and monopotassium), and therefore, the more P in the food, the more acid in the diet (the acidity of high-P food was common in several commercial rodent foods). In this work, the same neutral pH of 7.2 in the 3 fodders containing either 0.1%, 0.6%, or 1.2% was achieved through a combination of potassium phosphate dibasic and monosodium phosphate, similarly to what has been previously reported^35^ (see the “Materials and Methods” section). An acidic food in experimental animals caused by a high acidic Pi content can cause the state of a low level of metabolic acidosis and phosphaturia, even in the presence of an increased transport rate and the expression of several Na/Pi cotransporters.^36^ This apparent discrepancy can be explained by the acid pH-mediated reduction of the preferred substrate (HPO_4_^2−^) of the main Pi transporters in the kidney, NaPi2a and NaPi2c, in favor of the protonated dihydrogen phosphate ion (H_2_PO_4_^−^), which is the preferred substrate of the less active PiT1 and PiT2.^37^ In the small intestine, however, a higher transport rate is at a low pH, therefore favoring the absorption of H_2_PO_4_^−^.^12^, ^38^ Nevertheless, even if an acidic food could increase intestinal Pi absorption, this does not explain the changes in the intrinsic transport rate of duodenum and/or jejunum BBMV observed in vitro in previous works after acute adaptation to a high-Pi diet or when animals are fed for just 4 h per day.^11^, ^12^ Finally, endocrine disorders related to acidic food, ending in modest metabolic acidosis (eg, decreased levels of IGF-1 and PTH, mild hypothyroidism, hyperglucocorticoidism, increased calcitriol, etc.), could also have effects on intestinal Pi transport, given that most of such disorders have effects on Pi homeostasis. Nevertheless, the separate or combined effects of all these agents (if they are related) in causing a paradoxical increase in intestinal Pi transport, which has not been observed in this study, are difficult to understand.

Third, and finally, another unexpected finding was the rejection by normal animals to eat the high (1.2%) P food after the switch from a low to a high P food (Figure 2B). During the previous 5 d, all animals had eaten a similar amount of fodder every day, and therefore unpleasant-tasting food is an unlikely cause. In addition, the PTX rats not only exhibited the same food rejection when the food was switched, but every day they consumed less of the food containing 0.6% or 1.2% P than what they consumed of the 0.1% P food, as well as less than any of the 3 diets eaten by the sham-operated rats (Figure 5). The reduced consumption of P-rich fodder has been described previously in similar experiments of chronic and acute adaptations, but the intensity of the reduction was less than in our case.^8^, ^39^ In mice, a very high-P diet (2% P) impairs fatty acid metabolism, thereby reducing synthesis and oxidation, as well as spontaneous locomotor activity, and while a reduced appetite could also be involved, the food intake was not reported.^40^ The taste receptors T1R2 and T1R3 are involved in tasting phosphorus (including phosphates), and rodents prefer phosphate solutions over water.^41^ Previous works have shown an inverse relationship between plasma Pi concentration and Pi appetite in juvenile and adult rats.^42^, ^43^ It has also been suggested that the concentration of Pi in cerebrospinal fluid also plays a role because when the Pi concentration was experimentally raised in the third ventricle of rats being fed a low-Pi diet, the Pi-ingestive behavior of the animals seeking out a source of Pi was blunted.^44^

Our results also support these suggestions: Only a high Pi concentration in plasma correlates to food rejection. In fact, food rejection does not occur in normal rats eating a high-Pi diet, given that the hyperphosphatemia is transitory (after 4 h of feeding), and fasting rats show a normal Pi concentration independently of the food type (Figure 3C). In normal rats, food rejection only occurs with a switch from a low to a high P diet, meaning when a chronically low-P diet causes fast hyperphosphatemia (Figure 3C) as a consequence of the adaptation state: increased intestinal Pi transport rate, very low plasma concentrations of PTH and FGF23, and a high renal reabsorption rate. In the PTX rats, however, not only does the same switch from 0.1% P to 1.2% P cause a rejection of 1.2% P food, but it also causes chronic consumption of the 0.6% and 1.2% P diets (Figure 7A).

In conclusion, despite the progress in understanding not only the adaptation mechanisms of Pi homeostasis but also the sensing of Pi and the intestinal and renal adaptations of Pi, we are far from having satisfactory knowledge. Recently, an excellent work has related Pi sensing in the kidney to FGF23 production through the glycolytic synthesis of glycerol-3-phosphate.^45^ For now, we do not know how these data relate to the evidently predominant role of PTH over FGF23 in acute regulation to high Pi intake. Similarly, a lack of acute adaptation of renal Pi transport in the absence of PTH, even if the Pi transporters are regulated, the independent response of the intestine to these hormones while still adapted to the dietary Pi concentration, and the role of Pi fluxes from/toward intracellular stores besides the bone reservoir are important questions that need to be addressed.

## Data Availability

The data that support the findings of this study are available from the corresponding author upon reasonable request.
